# Development and Validation of a Simple-to-Use Nomogram for Predicting In-Hospital Mortality in Patients With Acute Heart Failure Undergoing Continuous Renal Replacement Therapy

**DOI:** 10.3389/fmed.2021.678252

**Published:** 2021-11-03

**Authors:** Luyao Gao, Yuan Bian, Shengchuan Cao, Wentao Sang, Qun Zhang, Qiuhuan Yuan, Feng Xu, Yuguo Chen

**Affiliations:** ^1^Department of Emergency Medicine, Qilu Hospital of Shandong University, Jinan, China; ^2^Shandong Provincial Clinical Research Center for Emergency and Critical Care Medicine, Chest Pain Center, Institute of Emergency and Critical Care Medicine of Shandong University, Qilu Hospital of Shandong University, Jinan, China; ^3^Key Laboratory of Emergency and Critical Care Medicine of Shandong Province, Key Laboratory of Cardiopulmonary-Cerebral Resuscitation Research of Shandong Province, Shandong Provincial Engineering Laboratory for Emergency and Critical Care Medicine, Qilu Hospital of Shandong University, Jinan, China; ^4^The Key Laboratory of Cardiovascular Remodeling and Function Research, The State and Shandong Province Joint Key Laboratory of Translational Cardiovascular Medicine, Chinese Ministry of Education, Chinese Ministry of Health and Chinese Academy of Medical Sciences, Qilu Hospital of Shandong University, Jinan, China

**Keywords:** acute heart failure, continuous renal replacement therapy, nomogram, prognostic model, mortality

## Abstract

**Background:** Patients with acute heart failure (AHF) who require continuous renal replacement therapy (CRRT) have a high risk of in-hospital mortality. It is clinically important to screen high-risk patients using a model or scoring system. This study aimed to develop and validate a simple-to-use nomogram consisting of independent prognostic variables for the prediction of in-hospital mortality in patients with AHF undergoing CRRT.

**Methods:** We collected clinical data for 121 patients with a diagnosis of AHF who underwent CRRT in an AHF unit between September 2011 and August 2020 and from 105 patients in the medical information mart for intensive care III (MIMIC-III) database. The nomogram model was created using a visual processing logistic regression model and verified using the standard method.

**Results:** Patient age, days after admission, lactic acid level, blood glucose concentration, and diastolic blood pressure were the significant prognostic factors in the logistic regression analyses and were included in our model (named D-GLAD) as predictors. The resulting model containing the above-mentioned five factors had good discrimination ability in both the training group (C-index, 0.829) and the validation group (C-index, 0.740). The calibration and clinical effectiveness showed the nomogram to be accurate for the prediction of in-hospital mortality in both the training and validation cohort when compared with other models. The in-hospital mortality rates in the low-risk, moderate-risk, and high-risk groups were 14.46, 40.74, and 71.91%, respectively.

**Conclusion:** The nomogram allowed the optimal prediction of in-hospital mortality in adults with AHF undergoing CRRT. Using this simple-to-use model, the in-hospital mortality risk can be determined for an individual patient and could be useful for the early identification of high-risk patients. An online version of the D-GLAD model can be accessed at https://ahfcrrt-d-glad.shinyapps.io/DynNomapp/.

**Clinical Trial Registration:** www.ClinicalTrials.gov, identifier: NCT0751838.

## Introduction

Acute heart failure is life-threatening and one of the most common causes of hospitalization worldwide. It is characterized by a high risk of in-hospital mortality and re-hospitalization, which may result from acute myocardial dysfunction (ischemia, inflammation, or toxicity), arrhythmia, uncontrolled hypertension, non-adherence to medication/diet, or volume overload, and requires timely treatment (1). Patients with acute heart failure (AHF) require admission to intensive care units (ICUs) and are usually critically ill with multiorgan failure, in which the kidneys are most frequently involved (2). The goals of the treatment for AHF in the ICU are to improve hemodynamic stability and organ perfusion, alleviate symptoms, and limit cardiac and renal damage (1), which can be achieved by continuous extracorporeal blood purification, known as continuous renal replacement therapy (CRRT) (3). The CRRT can mimic urine output by slowly and continuously removing the plasma water of the patient (4) and achieving accurate volume control and hemodynamic stability (5). The 2016 European Society of Cardiology guidelines recommended the consideration of renal replacement therapy (RRT) in patients with AHF with refractory volume overload and acute kidney injury (AKI) (1).

Continuous renal replacement therapy is the most commonly used mode of renal replacement therapy (RRT) and has been used increasingly on patients with AHF in the ICU in recent years (5). The Acute Heart Failure Global Survey of Standard Treatment (ALARM-HF) study showed that the in-hospital mortality rate of the patients with AHF in the ICU was ~17.8% (6), which was three times higher than those in the general ward. However, when an indicator for CRRT is confirmed in critically ill patients, the mortality rate is already up to 45–62.1% (7–9), which is twice that in patients with AHF in the ICU. Therefore, there is a need for an early scoring model or screening system that can help clinicians to intervene rapidly and ameliorate the disease outcome in patients with AHF undergoing CRRT, who are at high risk of mortality. The tools most widely used to predict mortality in critically ill patients are the Acute Physiology Assessment and Chronic Health Evaluation II (APACHE II), the Mortality Probability Model II, and the Simplified Acute Physiologic Score II (SAPS II) (10–12). However, the variables included in these scoring systems are too complex and inconvenient for routine use. The Modified Early Warning Score (MEWS) and SUPER score, SpO_2_, urine volume, pulse, emotional state, and respiratory rate are more concise than the APACHE II or SAPS II and can be used for the early warning of the onset of AHF in at-risk patients (13, 14). The APACHE II, SAPS II, and MEWS scores have some predictive value for the risk of death but their ability to predict in-hospital mortality in patients with AHF receiving CRRT is not known. To the best of our knowledge, there are no specialized scores or models that can predict in-hospital mortality in these patients.

This study aimed to develop and validate a simple-to-use nomogram model consisting of independent prognostic variables for the prediction of in-hospital mortality in adults with AHF undergoing CRRT. Meanwhile, the effectiveness of APACHE II, SAPS II, and MEWS in predicting the in-hospital mortality of AHF patients receiving CRRT was verified, and the most suitable model was selected and compared with the nomogram model to be widely popularized and applied.

## Materials and Methods

The study was approved by the Ethics Committee of the Qilu Hospital of Shandong University (approval number KYLL-202011-114).

### Data Source

The patient data used in this study was sourced from two databases. The first database was the Acute Heart Failure Unit (AHFU) at the Qilu Hospital of Shandong University, which opened on August 5, 2014, and was the first AHFU in China to advocate the concept of “early warning, early intervention” under the guidance of the SUPER score. Using this score, the onset of AHF can be predicted 2–6 h earlier than previously, and the rate of in-hospital mortality was decreased by more than 10% from 2012 to 2014 (13). The second was the medical information mart for intensive care III (MIMIC-III, version 1.4) database, a freely accessible, single-center, large online international database, which is approved by the institutional review boards of the Massachusetts Institute of Technology and Beth Israel Deaconess Medical Center (15) and contains data collected from more than 38,000 adults between 2001 and 2012. All the data from the MIMIC-III database were extracted by one of the investigators (Luyao Gao) after the completion of the collaborative institutional training initiative (CITI) program course with certification (ID 36599230).

### Study Population and Design

Patients with a diagnosis of AHF who underwent CRRT in the AHFU and those whose data were included in the MIMIC-III database were eligible for inclusion in the study. Patients who died before CRRT and those with missing information on the primary endpoint events were excluded. The eligible patients were randomly (7:3) allocated to the training cohort (*n* = 159) or the validation cohort (*n* = 67).

All the patients were categorized according to whether they were survivors or non-survivors at the time of discharge from the hospital. In principle, the time point of the clinical data collection of our model was when the physicians decided to initiate CRRT on the patients with AHF; the variables adopted were the newest ones we can acquire before the CRRT. If CRRT was needed to be initiated on the patient upon admission, we can refer to the laboratory reports in the emergency rooms or junior hospitals. An extensive list of baseline variables related to in-hospital mortality was identified (Table 1). The interval between the admission to the hospital and the start of the CRRT was also named days after the admission. For the study, in-hospital mortality was defined as all-cause mortality. We then developed novel clinical prediction models to predict the risk of in-hospital mortality.

**Table 1:** The baseline characteristics of the survivor cohort and the non-survivor cohort.

	**Overall**	**Non-Survivor**	**Survivor**	***P***
***N***	**226**	**98**	**128**	
Sex = Male (%)	123 (54.4)	56 (57.1)	67 (52.3)	0.56
Age (%)				<0.001
<45	23 (10.2)	4 (4.1)	19 (14.8)	
>70	98 (43.4)	60 (61.2)	38 (29.7)	
45~70	105 (46.5)	34 (34.7)	71 (55.5)	
Non-DM (%)	113 (50)	45 (45.9)	68 (53.1)	0.347
Non-HP (%)	123 (54.4)	50 (51.0)	73 (57)	0.445
Non-CAD (%)	105 (46.5)	39 (39.8)	66 (51.6)	0.105
Non-CKD (%)	94 (41.6)	48 (49)	46 (35.9)	0.066
Non-DN (%)	158 (69.9)	68 (69.4)	90 (70.3)	0.997
Non-CPR (%)	205 (90.7)	84 (85.7)	121 (94.5)	0.042
MV (%)				0.079
Without MV	99 (43.8)	35 (35.7)	64 (50)	
IMV	83 (36.7)	43 (43.9)	40 (31.2)	
non IMV	44 (19.5)	20 (20.4)	24 (18.8)	
Temperature = 35–38.5°C (%)	203 (89.8)	92 (93.9)	111 (86.7)	0.123
Heart Rate (%)				0.339
<90 beats/min	122 (54)	54 (55.1)	68 (53.1)	
>140 beats/min	7 (3.1)	1 (1.0)	6 (4.7)	
90–140 beats/min	97 (42.9)	43 (43.9)	54 (42.2)	
Respiration (%)				0.625
<20 breaths/min	103 (45.6)	42 (42.9)	61 (47.7)	
≥30 breaths/min	15 (6.6)	8 (8.2)	7 (5.5)	
20–30 breaths/min	108 (47.8)	48 (49.0)	60 (46.9)	
SBP >120 mmHg (%)	81 (35.8)	26 (26.5)	55 (43.0)	0.016
DBP >60 mmHg (%)	107 (47.3)	35 (35.7)	72 (56.2)	0.003
MAP ≥70 (%)	149 (65.9)	58 (59.2)	91 (71.1)	0.084
SPO2 (%)				0.016
≤ 94	39 (17.3)	25 (25.5)	14 (10.9)	
≥99	115 (50.9)	44 (44.9)	71 (55.5)	
94–98	72 (31.9)	29 (29.6)	43 (33.6)	
UA (%)				0.143
≤ 30	136 (60.2)	62 (63.3)	74 (57.8)	
≥50	48 (21.2)	15 (15.3)	33 (25.8)	
30–50	42 (18.6)	21 (21.4)	21 (16.4)	
WBC > 10*10^∧^9/L (%)	117 (51.8)	53 (54.1)	64 (50)	0.635
NEU% >75% (%)	141 (62.4)	67 (68.4)	74 (57.8)	0.138
Hemoglobin >90 g/L (%)	121 (53.5)	55 (56.1)	66 (51.6)	0.585
Platelet >130*10^∧^9/L (%)	151 (66.8)	58 (59.2)	93 (72.7)	0.047
Potassium 3.5–5.5 mmol/L (%)	169 (74.8)	68 (69.4)	101 (78.9)	0.139
Sodium = 137–147 mmol/L (%)	93 (41.2)	35 (35.7)	58 (45.3)	0.188
Calcium = 2.0–2.6 mmol/L (%)	130 (57.5)	51 (52)	79 (61.7)	0.186
ALT >40 U/L (%)	87 (38.5)	45 (45.9)	42 (32.8)	0.062
AST >60 U/L (%)	101 (44.7)	53 (54.1)	48 (37.5)	0.019
Creatinine >430 umol/L (%)	85 (37.6)	22 (22.4)	63 (49.2)	<0.001
BUN >20 mmol/L (%)	138 (61.1)	55 (56.1)	83 (64.8)	0.232
Blood glucose >10 mmol/L (%)	68 (30.1)	38 (38.8)	30 (23.4)	0.019
Lactic acid>1.8 mmol/L (%)	92 (40.7)	50 (51.0)	42 (32.8)	0.009
NT-proBNP [n (%), median (IQR)]	126 (55.75%), 20725.00 (10018.25, 35,000)	70 (71.43%), 27008.50 (10766.50, 35,000)	56 (43.75%), 19,843 (8843.25, 35,000)	0.423
Troponin I [n (%), median (IQR)]	52 (23%), 0.23 (0.03, 1.32)	32 (32.65), 0.30 (0.04, 1.75)	20 (15.63%), 0.08 (0.03, 1.20)	0.402
Troponin T [n (%), median (IQR)]	85 (37.61%), 0.22 (0.09, 0.98)	38 (38.78%), 0.18 (0.06, 0.62)	47 (36.72%), 0.34 (0.12, 1.19)	0.164
Days after admission before CRRT (%)				<0.001
≤ 3d	107 (47.3)	30 (30.6)	77 (60.2)	
>10d	44 (19.5)	33 (33.7)	11 (8.6)	
4–10d	75 (33.2)	35 (35.7)	40 (31.2)	
MEWS (mean ± SD)	3.16± 2.02	3.54 ±1.93	2.88 ±2.05	0.014
SUPER Score (mean ± SD)	3.45 ±1.59	3.73 ±1.60	3.23 ±1.55	0.019

*ALT, alanine transaminase; aST, Aspartate aminotransferase; DM, diabetes mellitus; BUN, blood urea nitrogen; CAD, coronary artery disease; CKD, chronic kidney disease; CPR, cardiopulmonary resuscitation; DBP, diastolic blood pressure; DN, diabetic nephropathy; IMV, invasive mechanical ventilation; IQR, interquartile range; MAP, mean arterial pressure; MEWS, modified early warning score; MV, mechanical ventilation; NEU%, neutrophils ratio; NT-proBNP, N-terminal precursor B-type diuretic peptide; SBP, systolic blood pressure; WBC, white blood cell*.

If the proportion of the missing values was <5%, it was replaced with mean or median values; if the proportion was more than 5%, the missing values were imputed using multiple linear regression. Some values, such as those for the N-terminal precursor B-type diuretic peptide (NT-proBNP) and troponin, for which the missing proportion was over 60%, were only analyzed using the existing data.

### Statistical Analysis

The categorical variables were expressed as a percentage and compared using the chi-squared test or Fisher's exact test. The continuous variables were summarized as the mean and SD or the median [interquartile range (IQR)] and compared using the *t*-test and Kruskal–Wallis test, respectively.

The magrittr package was used to randomly divide the eligible patients into the training and validation cohort. Univariate logistic regression analyses were performed to determine the independent risk factors for the in-hospital all-cause deaths in the training cohort. The odds ratios (ORs) and 95% CIs were calculated for these variables to quantify the strength of the associations. All the variables that showed a relationship with in-hospital mortality in the univariate analysis or were considered clinically relevant were candidates for the stepwise multivariate analysis in the training cohort. A nomogram model, produced using the rms package, was formulated based on the results for the independent risk factors identified in the multivariate logistic regression. Based on the nomogram model, the total scores and prediction of the in-hospital mortality risk for each patient were added for each eligible variable and then converted to predicted probabilities in both the training and validation cohorts.

To evaluate the ability of the model to predict in-hospital mortality, we first calculated the calibration of the model using 1,000 bootstrap samples to decrease the overfit bias. The Hosmer–Lemeshow test was used to evaluate the goodness of the fit. Second, the Harrell concordance index (C-index) and receiver-operating characteristic curve (ROC) analysis were used to evaluate the predictive performance and discrimination ability of the nomogram. A ROC analysis was used to calculate the optimal cutoff values, which were determined by maximizing the Youden index. Third, the clinical effectiveness of the resulting model was evaluated by a decision curve analysis (DCA) and clinical impact curve (CIC), which is a method for evaluating diagnostic or prognostic tools that potentially have advantages over others (16, 17). The increase in the discriminative value of the MEWS and the resulting model for mortality were assessed using the Net Reclassification Index (NRI).

All statistical analyses were performed using STATA version 15.0 (StataCorp LLC, College Station, TX, USA) and R language software (v4.0.3, http://www.r-project.org/). The packages used in the study were tableone, foreign, rms, broom, magrittr, pROC, rmda, blorr, PredictABEL, ResourceSelection, and ggplot2. The *p* < 0.05 was considered statistically significant.

## Results

### Characteristics of the Study Population

A total of 226 patients with AHF who underwent CRRT during the study period were enrolled and grouped according to whether they were discharged from the hospital as non-survivors (*n* = 98, 43.4%) or survivors (*n* = 128, 56.6%). The mortality rate in the validation cohort was 46.3 and 42.1% in the training cohort; both these values were lower than the previously reported rate of 58.1% (7). The demographics and clinical characteristics of the patients in the non-survivors and survivors' cohort are summarized in Table 1. Compared with the survivors, the non-survivors were older and more likely to receive more cardiopulmonary resuscitation (CPR), have lower systolic blood pressure (SBP) and diastolic blood pressure (DBP), lower creatinine and platelet levels, lower oxyhemoglobin saturation (SpO_2_), more likely to have higher aspartate aminotransferase (AST), blood glucose, and lactic acid, and to have a longer interval between the admission to the hospital and starting the CRRT. The NT-proBNP was only collected in 126 patients as more than half of the patients had renal insufficiency and nearly 30% of the test value of the patients exceeds the upper limit (35,000 ng/L). The troponin was also missing up to 50% of the data, so both values were not included in the model building. The demographics and clinical characteristics of the training cohort are detailed in Supplementary Table 1. There was no significant difference in any of the clinicopathological data except for the potassium level and SUPER score, which were more abnormal in the validation cohort and may explain why the mortality was slightly higher in that cohort. The details can be found in Supplementary Table 2.

### Logistic Regression Analyses

All the variables used in these analyses were based on retrospectively obtained data. The results of the univariate logistic analysis are presented in Table 2. In addition to the variables that were statistically significant in univariate analysis (*p* < 0.05), namely, age, need for CPR, SBP, DBP, SpO_2_, urine volume, AST, creatinine, blood glucose, and lactic acid levels, and the interval between admission to the hospital and starting CRRT, the variables considered as clinically related to in-hospital mortality, such as mechanical ventilation (MV), mean arterial pressure (MAP), and SUPER score, were candidates for the stepwise multivariate analysis in the training cohort.

**Table 2:** Univariate logistic regression analysis of in-hospital mortality in the training cohort.

**Variable**	**OR**	**95% CI**		***P-*value**
Sex Female vs. Male	0.95	0.50	1.79	0.8723
**Age**				
<45	Ref.			
45–70	2.46	0.73	11.30	0.1826
>70	8.89	2.63	41.13	0.0013
DM	1.53	0.82	2.90	0.1852
Hypertension	1.17	0.62	2.21	0.6268
CAD	1.62	0.86	3.08	0.1396
CKD	0.76	0.40	1.44	0.3943
DN	1.12	0.57	2.20	0.7475
CPR	3.41	1.06	13.08	0.0492
**MV**				
Without MV	Ref.			
non-IMV	1.41	0.60	3.28	0.4304
IMV	1.81	0.89	3.73	0.1056
**T (** **°** **C)**				
35–38.5	Ref.			
<35 or >38.5	0.43	0.09	1.52	0.2223
**Heart Rate (beats/min)**				
<90	Ref.			
90–140	0.87	0.46	1.66	0.6820
>140	0.00	NA	0.00	0.9891
**Respiration (beats/min)**				
<20	Ref.			
20–30	0.95	0.49	1.81	0.8686
≥30	1.08	0.25	4.38	0.9189
**SBP (mmHg)**				
>120	Ref.			
≤ 120	2.50	1.28	5.00	0.0082
**DBP (mmHg)**				
>60	Ref.			
≤ 60	3.04	1.59	5.95	0.0009
**MAP (mmHg)**				
>70	Ref.			
≤ 70	1.91	0.98	3.78	0.0595
**SpO**_**2**_ **(%)**				
≥99	Ref.			
95–98	0.83	0.40	1.71	0.6176
≤ 94	2.84	1.11	7.72	0.0328
**Urine volume (ml/h)**				
≥50	Ref.			
30–50	2.38	0.86	6.91	0.1013
≤ 30	2.59	1.13	6.41	0.0299
**WBC (10** ^**∧**^ **9/L)**				
≤ 10	Ref.			
>10	1.52	0.81	2.88	0.1965
**NEU% (%)**				
≤ 75	Ref.			
>75	1.21	0.63	2.32	0.5737
**HGB (g/L)**				
>90	Ref.			
≤ 90	0.63	0.33	1.19	0.1540
**Platelet (10** ^**∧**^ **9/L)**				
>130	Ref.			
≤ 130	1.63	0.84	3.17	0.1513
**Potassium (mmol/L)**				
3.5–5.5	Ref.			
<3.5 or >5.5	1.89	0.87	4.13	0.1078
**Sodium (mmol/L)**				
137–147	Ref.			
<137 or >147	1.35	0.71	2.59	0.3600
**Calcium (mmol/L)**				
2.0–2.6	Ref.			
<2.0 or >2.6	1.12	0.59	2.11	0.7268
**ALT (U/L)**				
≤ 40	Ref.			
>40	1.35	0.70	2.58	0.3685
**AST (U/L)**				
≤ 60	Ref.			
>60	2.18	1.15	4.18	0.0179
**Creatinine (umol/L)**				
≤ 430	Ref.			
>430	0.33	0.16	0.65	0.0020
**BUN (mmol/L)**				
≤ 20	Ref.			
>20	0.90	0.47	1.70	0.7356
**Blood glucose (mmol/L)**				
≤ 10	Ref.			
>10	2.78	1.38	5.72	0.0048
**Lactic acid (mmol/L)**				
≤ 1.8	Ref.			
>1.8	2.68	1.40	5.19	0.0031
**Days after admission before CRRT**				
≤ 3d	Ref.			
4–10d	2.59	1.24	5.53	0.0123
>10d	6.84	2.78	18.08	<0.0001
MEWS	1.11	0.95	1.30	0.1855
SUPER score	1.23	1.00	1.53	0.0514

*ALT, Alanine transaminase; AST, Aspartate aminotransferase; DM, diabetes mellitus; BUN, blood urea nitrogen; CAD, coronary artery disease; CI, confidence interval; CKD, chronic kidney disease; CPR, cardiopulmonary resuscitation; DBP, diastolic blood pressure; DN, diabetic nephropathy; IMV, invasive mechanical ventilation; MAP, mean arterial pressure; MEWS, modified early warning score; MV, mechanical ventilation; NEU%, neutrophils ratio; OR, odds Ratio; SBP, systolic blood pressure; WBC, white blood cell*.

**Figure 1:**
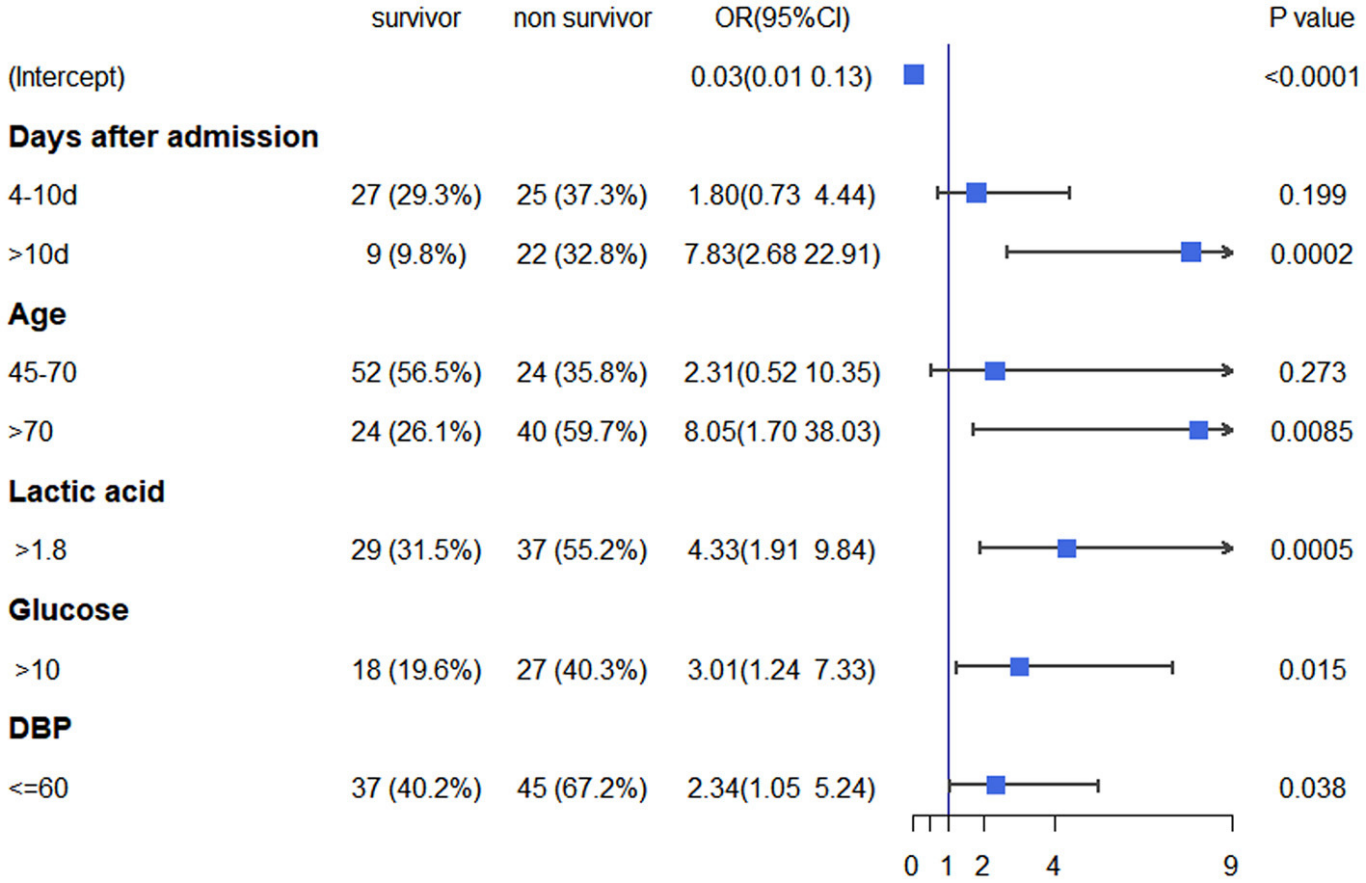
Multivariate logistic regression analysis of in-hospital mortality based on pre-CRRT data in the training cohort. CRRT, continuous renal replacement therapy.

The analyses showed that the factors independently associated with in-hospital mortality were age (45–70 years vs. <45 years [OR 2.31, 95% CI 0.52–10.35; *p* = 0.273] and >70 years vs. <45 years [OR 8.25, 95% CI 1.7–38.03; *p* = 0.0085]), days after admission (4–10 days vs. <3 days [OR 2.31, 95% CI 0.52–10.35; *p* = 0.199] and >10 days vs. <3 days [OR 7.83, 95% CI 2.68–22.91; *p* = 0.002]), lactic acid (OR 4.33, 95% CI 1.91–9.84; *p* = 0.0005), blood glucose (OR 3.01, 95% 1.24–7.33; *p* = 0.015), and DBP (OR 2.34, 95% CI 1.05–5.24; *p* = 0.038; Figure 1).

### Nomogram Model and Webserver

**Figure 2:**
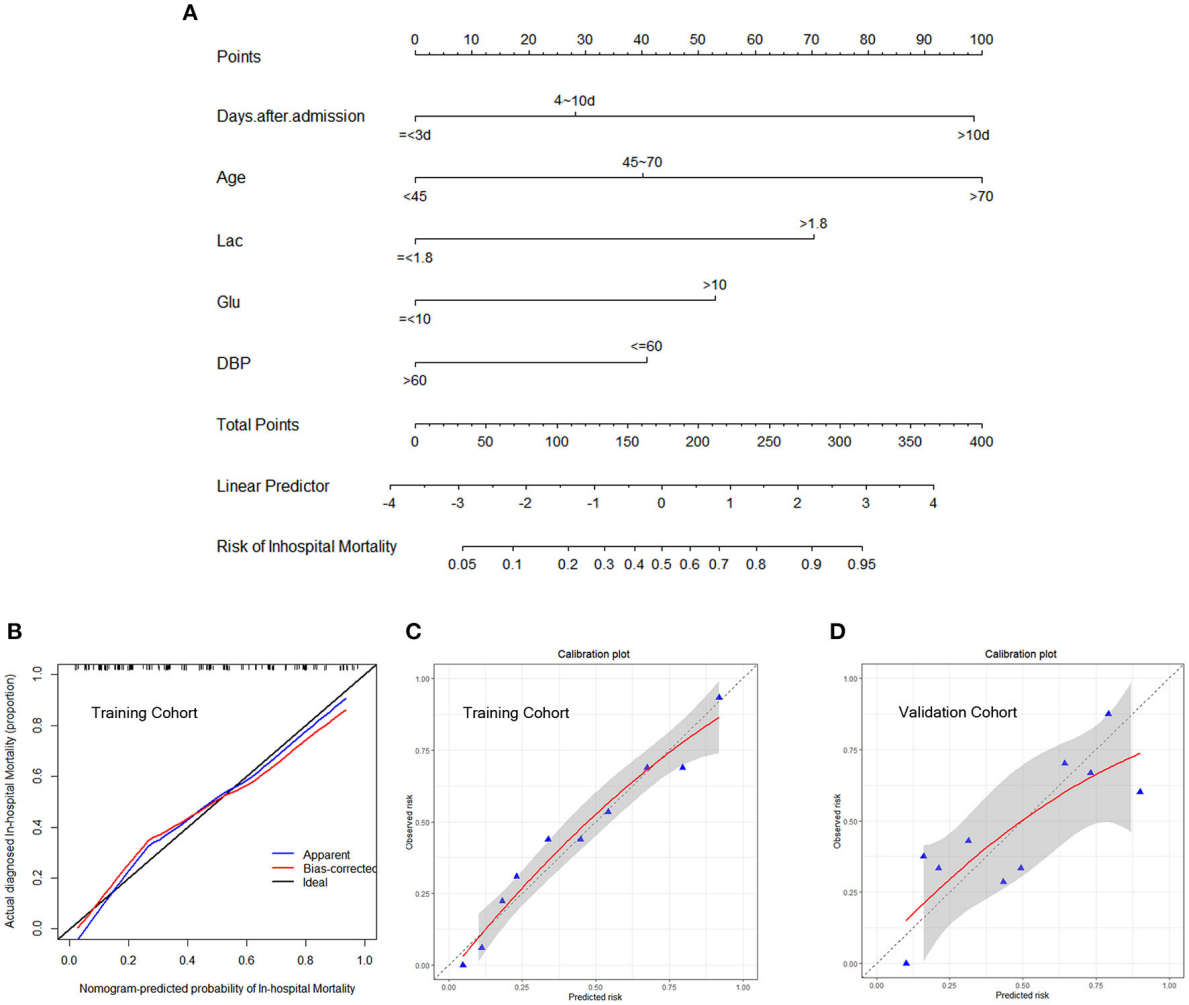
Development predicting nomogram model. **(A)** The nomogram includes significant clinical characteristics for predicting in-hospital mortality in AHF patients undergoing CRRT. To estimate the in-hospital mortality rate of an individual patient, the value of each included significant clinical characteristic is acquired and divided into the different groups, followed by a line drawn straightly downward to determine the points. The sum of these five numbers is located at the Total Points axis, then a line is drawn downward to the risk of in-hospital mortality axes to determine the likelihood of in-hospital mortality. The calibration curve of the nomogram model for predicting in-hospital mortality was internally validated using the bootstrap validation method **(B)** and Hosmer–Lemeshow test **(C)** in the training cohort and externally validated using the Hosmer–Lemeshow test **(D)** in the validation cohort. The nomogram-predicted probability of in-hospital mortality is plotted on the x-axis, and the actual in-hospital mortality is plotted on the y-axis. The gray area both in c and d represents a 95% confidence interval. AHF, acute heart failure; CRRT, continuous renal replacement therapy; DBP, diastolic blood pressure; Lac, lactic acid.

The independently associated risk factors (age, days after admission, lactic acid, blood glucose, and DBP) were used to form an in-hospital mortality risk estimation nomogram (Figure 2A). To allow clinicians to use this tool, which we have named the D-GLAD model, more conveniently and easily, we used the DynNom package and shinyapps (https://www.shinyapps.io) to build an online webserver (https://ahfcrrt–d-glad.shinyapps.io/DynNomapp/), which can show the individualized prediction dynamically by inputting the clinical features. Clinicians and researchers can predict in-hospital mortality by reading the output figures and tables generated by the webserver (Supplementary Figure 1).

### Validation of the Nomogram Model

**Figure 3:**
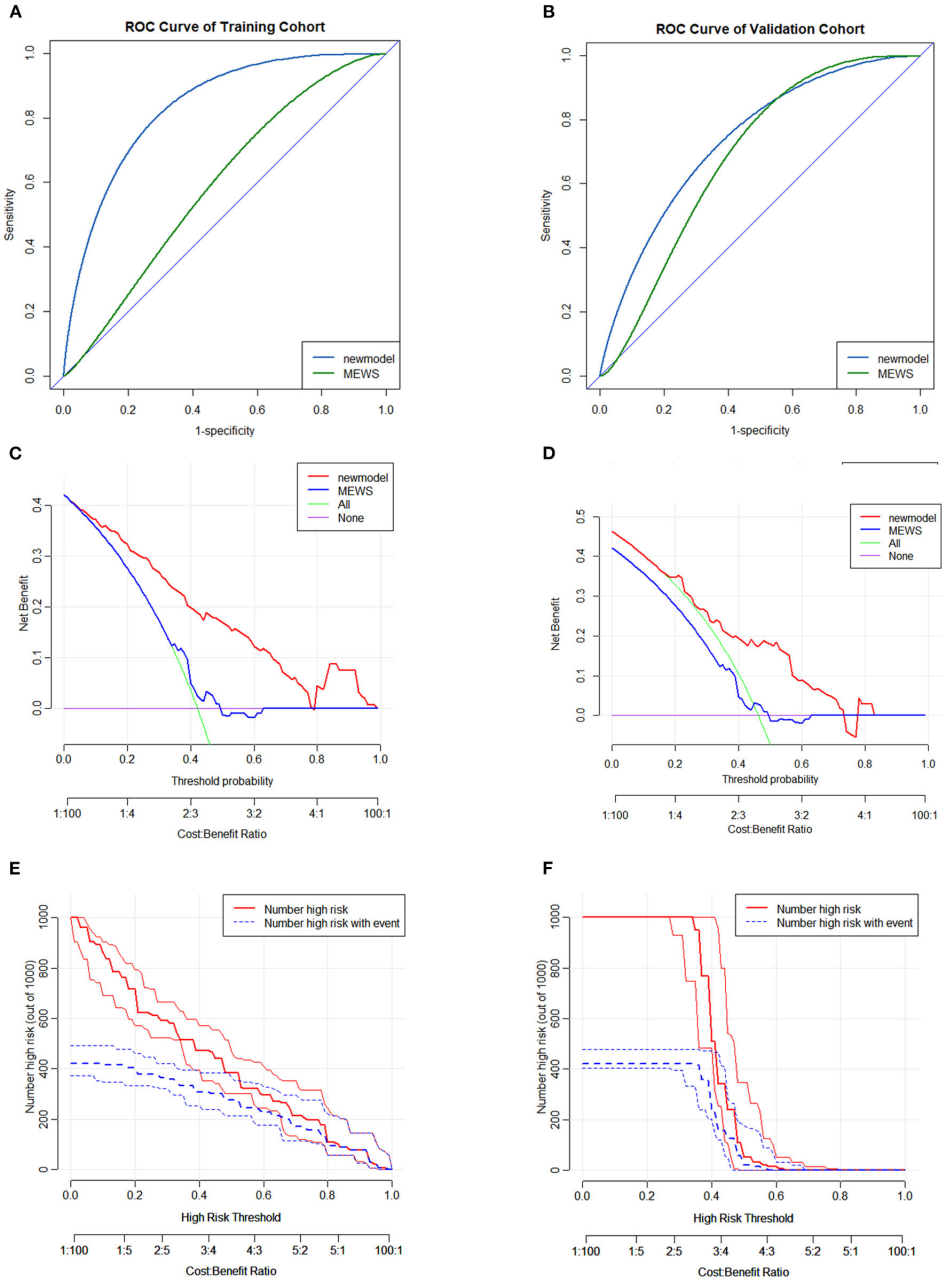
Discrimination and clinical effectiveness validation of predicting nomogram model. The ROC analyses of in-hospital mortality of the nomogram model and MEWS in the training cohort **(A)** and validation cohort **(B)**. DCA curve for in-hospital mortality in the training cohort **(C)** and validation cohort **(D)**. CIC for in-hospital mortality in nomogram model **(E)** and MEWS **(F)** in the training cohort. CIC, clinical impact curve; DCA, decision curve analyses; MEWS, Modified Early Warning Score; ROC, receiver operating characteristic curve.

The calibration of the nomogram model was internally validated using the bootstrap method and the Hosmer–Lemeshow test (*p* = 0.868); the externally validated Hosmer–Lemeshow test (*p* = 0.1043), showed good agreement with the concordance of the nomogram (Figures 2B–D). The nomogram demonstrated good accuracy in estimating the risk of the in-hospital all-cause mortality, with an unadjusted C-index of 0.829 (95% CI 0.767–0.891) in the training cohort, which was significantly higher than that of the MEWS (C-index 0.578, 95% CI 0.491–0.666; *p* < 0.001; Figure 3A). The C-index for the D-GLAD model was 0.740 (95% CI 0.620–0.860) in the validation cohort and was 0.685 (95% CI 0.558–0.813) for the MEWS. Although there was no statistically significant difference in the C-index value between the D-GLAD model and MEWS, the value was much larger for D-GLAD (Figure 3B).

Compared with the MEWS, the results of the DCA and the CIC demonstrated that the D-GLAD model had good clinical effectiveness in both the training and validation cohorts (Figures 3C–F). All the results indicated that the accuracy, discrimination ability, and clinical effectiveness of the D-GLAD model were superior to those of the MEWS.

### D-GLAD in AHFU and MIMIC-III

Only the APACHE II data could be extracted from the AHFU database and only the SAPS II data could be extracted from the MIMIC-III database.

**Figure 4:**
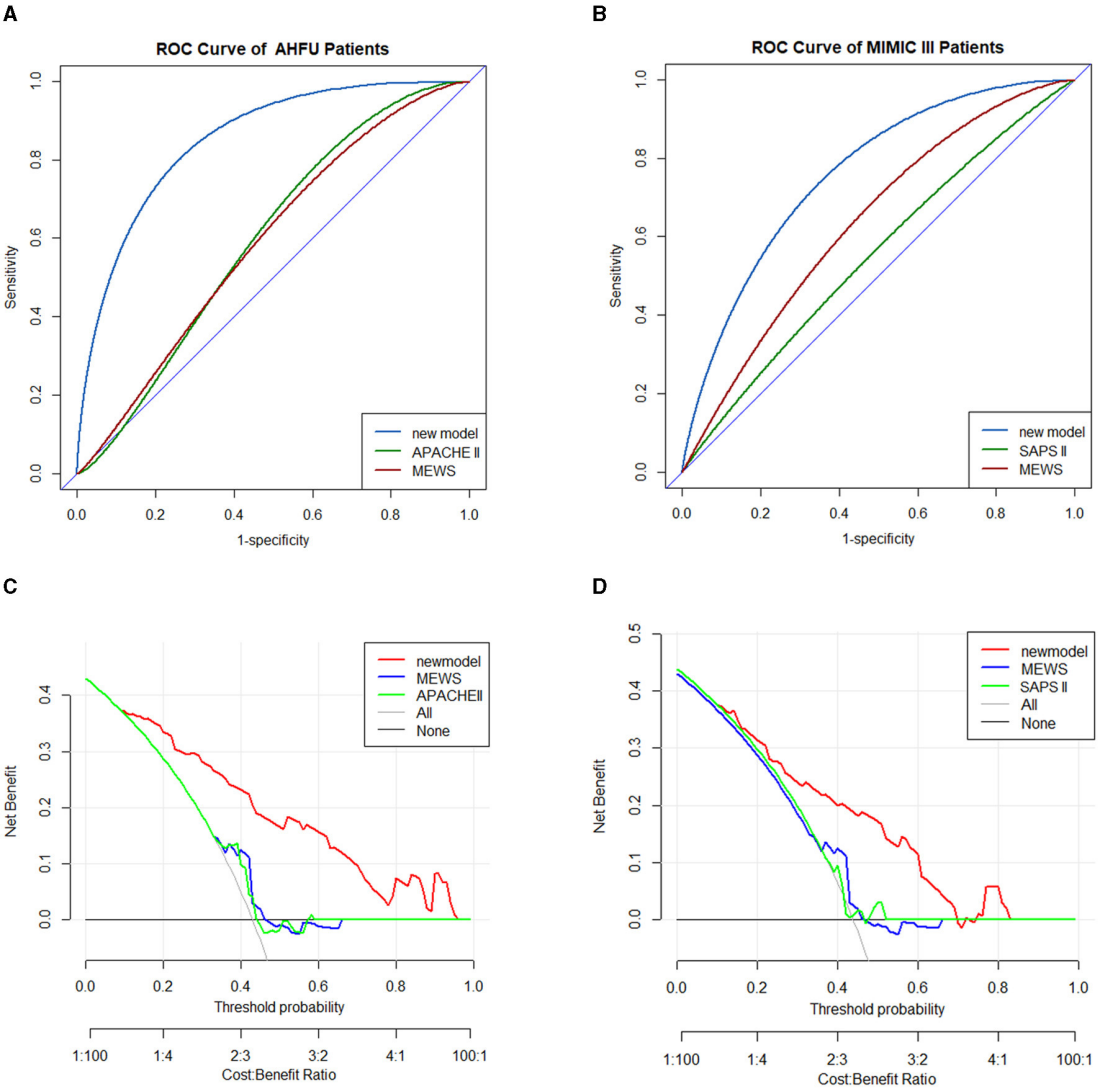
Validation of predicting nomogram model in AHFU and MIMIC III patients. The ROC analyses of in-hospital mortality of the nomogram model and MEWS in the AHFU cohort **(A)** and MIMIC III cohort **(B)**. DCA curve for in-hospital mortality in the AHFU cohort **(C)** and MIMIC III cohort **(D)**. AHFU, acute heart failure unit; DCA, decision curve analyses; MEWS, Modified Early Warning Score; MIMIC III, medical information mart for intensive care III; ROC, receiver operating characteristic curve.

In the AHFU cohort, the C-index for the D-GLAD model was 0.845 (95% CI 0.779–0.912), which was significantly different (*p* < 0.001) from that of the APACHE II (0.579, 95% CI 0.478–0.680) and MEWS (0.604, 95% CI 0.505–0.704; Figure 4A). The clinical effectiveness was similar for the MEWS and APACHE II but was much better for the D-GLAD model (Figure 4B).

In the MIMIC-III cohort, the C-index for the D-GLAD model was 0.759 (95% CI 0.667–0.851), which was significantly different (*p* = 0.0025) from that of the SAPS II (0.535, 95% CI 0.422–0.647) but not from that of the MEWS (0.618, 95% CI 0.512–0.724, *p* = 0.0564; Figure 4C). The clinical effectiveness of the D-GLAD model was much better than that of the MEWS and SAPS II (Figure 4D).

### D-GLAD Model Predict In-Hospital Mortality

The ROC curve showed that the optimal cutoff value for predicting in-hospital mortality was 0.266, with a corresponding total nomogram score of ~122. We used the prediction and total score for the risk stratification, where <125, 125–170, and >170 corresponded to low risk, moderate risk, and high risk, respectively. The NRI showed that the resulting model had better prognostic discrimination ability than the MEWS for the in-hospital mortality in both the training cohort (NRI, 59.14%; *p* < 0.001) and the validation cohort (NRI, 23.84%; *p* = 0.187). Using the D-GLAD model, the in-hospital mortality rate was ~14.46% in the low-risk group and up to 40.74% in the moderate-risk group. The in-hospital mortality was up to 71.91% in the high-risk group using the D-GLAD model and up to 65% in the extremely high-risk group using the SUPER score (Table 3). When used for screening high-risk patients, the sensitivity, specificity, positive predictive value, and negative predictive value were 73.5, 76.6, 57.2, and 87.2%, respectively, in the training cohort and 83.87, 44.44, 56.52, and 76.19% in the validation cohort.

**Table 3:** Predictive value of D-GLAD model and SUPER score for in-hospital mortality.

		**D-GLAD model**					
		**Low-risk <125**		**Moderate-risk 125–170**		**High-risk >170**	
SUPER Score	Low-risk	6.67%	(1/15)	50.00%	(2/4)	57.14%	(4/7)
	Moderate-risk	9.38%	(3/32)	39.13%	(9/23)	72.73%	(24/33)
	High-risk	13.79%	(4/29)	34.78%	(8/23)	75.00%	(30/40)
	Extremely high-risk	57.14%	(4/7)	75.00%	(3/4)	66.67%	(6/9)
	All	14.46%	(12/83)	40.74%	(22/103)	71.91%	(64/89)

## Discussion

In this study, we not only clarified the clinical features of patients with AHF undergoing CRRT and their risk factors for mortality but also developed and validated a nomogram model that could predict in-hospital mortality in these patients based on the data from the Qilu Hospital AHFU and MIMIC-III database. Patient age, days after admission, lactic acid level, blood glucose concentration, and DBP were the independent predictors of in-hospital mortality and were used to form a D-GLAD model that performs better than the APACHE II, SAPS II, and MEWS, and can help clinicians for the early screening of high-risk patients.

Acute heart failure is a severe disease with high mortality and hospital readmission rates and is characterized by the rapid onset or worsening of symptoms of heart failure, mostly associated with systemic congestion (18). Several observational studies in patients with AHF have demonstrated that fluid overload is independently associated with increased morbidity and mortality (19, 20). One reason was that patients with AHF are at risk of death not only from cardiovascular disease (CVD) but also from multiorgan failure, such as AKI. Acute kidney injury was more common in patients with AHF (nearly 24.3%), compared with those without AKI, and the risk of in-hospital mortality was more than 2-fold higher in patients with AKI (21). Continuous renal replacement therapy is the predominant RRT modality used for critically ill patients in ICUs (5) and can address congestion, reduce fluid overload, and maintain acid-base balance to improve the survival rate.

The predictors of in-hospital mortality in patients with AHF undergoing CRRT have been reported to include older age, lower SBP and DBP, and a decreased serum creatinine level (22), which is consistent with our research. We found that age was an undoubtedly hazardous factor for AHF in that the older the patient, the higher the mortality rate, which is consistent with previous research (23). The age-related structural and functional changes in the body, along with reduced compensatory capacity, are irreversible. Diastolic blood pressure and SBP also play an important role in AHF patients (24). The perfusion of the coronary arteries occurs during diastole, and when the DBP becomes too low to maintain the perfusion, the blood flow to the coronary arteries is reduced and the heart cannot obtain enough oxygen to function, which causes damage to the heart. Our results showed that a DBP ≤ 60 mmHg was an independent risk factor for in-hospital mortality, similar to previous studies in which intradialytic hypotension during the 1st h after initiation of CRRT has been identified as an independent predictor of high in-hospital mortality (25). However, this does not mean that a higher DBP is necessarily beneficial once hypertension does occur and the risk of stroke increases, so we advocate maintaining the DBP at 60–90 mmHg using vasoactive or antihypertensive agents or not.

The activation of the sympathetic nervous system (SNS), usually caused by cardiac output reduction in AHF patients, is one of the major neurohormonal mechanisms of the development or progression of AHF and promotes cardiomyocyte hypertrophy and fibrosis, impairing the diastolic and systolic functions of the heart (26). The activation of the SNS also causes the inhibition of glucose-stimulated insulin secretion and the increase of glucagon secretion *via* the α-receptor, caused hyperglycemia (27). In AHF patients, hyperglycemia may be the response to the danger and is a reflection of an activated SNS. Hyperglycemia in the hospitalized patients may be caused not only by the poor glycemic control in diabetes but also by a transient stress response to current disease states, named stress hyperglycemia (28). Hyperglycemia upon admission was independently associated with in-hospital and short-term mortality in AHF patients and was an independent predictor of 1-year mortality in non-diabetes Mellitus (DM) patients with AHF (27, 29). Several studies have consistently shown that relative hyperglycemia is more strongly associated with in-hospital mortality than absolute hyperglycemia in patients with diabetes (28, 30, 31). Blood glucose needs to maintain stability in the body, although our results only showed that a blood glucose ≤ 10 mmol/L was a positive factor. Hypoglycemia can lead to an insufficient energy supply to the brain and heart, which leads to neuropsychiatric symptoms, palpitations and tremors, and even comatose, sudden death, and other adverse events in severe cases. It was reasonable to control blood glucose in the range of 5–10 mmol/L or strictly 8–10 mmol/L to reduce the side effects of hyperglycemia and hypoglycemia.

Lactic acid is the mesostate of blood glucose and is produced mainly by glycolysis due to stress or hypoxia (for example, shock or arterial embolism) when the aerobic metabolism of blood glucose is reduced and then goes through glycolysis to produce energy. Breaking the balance between lactic acid production and elimination can promote lactate accumulation, called hyperlactatemia. In the setting of AHF, several mechanisms, such as peripheral hypoperfusion, low cardiac output, activation of AHF, hypoxemia, and liver or renal dysfunction (elimination lactate), can alter the lactate homeostasis (32). It is well-documented that elevated lactate levels and their continued elevation are useful for identifying high-risk patients and for predicting worse outcomes and the high risk of mortality in patients with AHF (33, 34). The elevated blood lactate acid, ≥2 mmol/L, predicted nearly 1.8-folds on 1-year mortality than low blood lactate acid (<2.5 mmol/L) (33). In our study, elevated lactate levels were a strong risk factor for death, which is consistent with the results of previous studies.

In patients with AHF with refractory volume overload and AKI, CRRT was recommended to alleviate the cardiac load and release condition. However, the time to initiate RRT in AHF remains controversial due to the lack of targeted research. Bart et al. compared ultrafiltration with diuretic-based therapy in patients with acute decompensated heart failure and worsened renal function, and found that the rates of death and rehospitalization did not differ significantly between the two treatments strategies (35). Our study showed that the interval between the admission in the hospital and starting CRRT was an independent predictor of in-hospital mortality, possibly because the early initiation of CRRT can allow the better control of metabolic abnormalities and other complications associated with increased mortality (36). This finding is consistent with previous reports. However, patients could be needlessly exposed to iatrogenic complications, such as hypotension, bleeding, infection, and hypothermia, which might explain why there was no statistically significant difference between 4–10 and <3 days in our study. When more than 10 days have elapsed before the initiation of CRRT, the patient mortality was higher because the conditions were mostly severe even after meticulous medical care. However, there was an inevitable selection bias in our research because those who died before meeting the criteria for initiation of CRRT or improved without the need for RRT were excluded (37). Follow-up data were not available for eligible patients in our study and could be investigated by clinicians or researchers of future studies.

We found that early treatment with intravenous loop diuretics (38), improved SBP, mechanical ventilation (39), and urine volume were associated with in-hospital mortality in AHF patients, and whether these variables could be used to improve the model is still unclear.

To our knowledge, the D-GLAD model is the first nomogram developed from data collected from more than one center that can be used to screen for patients with AHF at high risk of needing CRRT. Moreover, this is the first study to use the APACHE II score, SAPS II score, and MEWS to predict the risk of in-hospital mortality in patients with AHF receiving CRRT and to compare the results with those obtained using the D-GLAD model. However, the study had several limitations. First, we only recorded the in-hospital mortality of eligible patients and did not include further follow-up of survivors. A large, well-designed prospective study with a long-term follow-up is needed to validate and perfect the model. Second, we could only identify 226 patients who met the inclusion criteria; an addition of a greater number of eligible patients to the database will allow us to construct a more stable and accurate model. Third, we only analyzed patients who received CRRT, so whether the D-GLAD model can be used to guide the initiation of CRRT in patients who are hesitant to accept it is still unknown. Finally, this model is mostly based on the data from Caucasian and Asian populations, and the extent to which it can be adapted for use in other ethnic populations is unclear.

In conclusion, the D-GLAD model is the first nomogram to be derived from data obtained from more than one center (an AHFU database and the MIMIC-III database) and allowed the optimal prediction of in-hospital mortality in patients with AHF undergoing CRRT. The validation results in our training cohort and external cohort demonstrated that the nomogram performed well and had high accuracy, discrimination ability, and clinical effectiveness. Using this simple-to-use model, the risk of in-hospital mortality can be determined for an individual patient, which can be useful to guide the early screening of high-risk patients. Combined with the SUPER score, the D-GLAD model can more accurately assess the risk of in-hospital mortality in the AHF patients receiving CRRT.

## Data Availability Statement

The raw data supporting the conclusions of this article will be made available by the authors, without undue reservation.

## Ethics Statement

The studies involving human participants were reviewed and approved by the Ethics Committee of Qilu Hospital of Shandong University (KYLL-202011-114). Written informed consent for participation was not required for this study in accordance with the national legislation and the institutional requirements.

## Author Contributions

LG and YB conception of the study and writing of the manuscript. LG collected the data from the MIMIC III. WS and QZ collection of the data from the Qilu Hospital Acute Heart Failure Unit and analysis of all data. QY and SC revision of the manuscript. FX and YC critical revision of the manuscript. All authors contributed to the article and approved the submitted version.

## Funding

This study was funded by the Clinical Research Center of Shandong University (Nos. 2020SDUCRCC018 and 2020SDUCRCA006) and the National Natural Foundation of China (81801942). It was also supported by the National Key R&D Program of China (2020YFC1512700, 2020YFC1512705, 2020YFC1512703, and 2020YFC0846600), National S&T Fundamental Resources Investigation Project (2018FY100600 and 2018FY100602), Taishan Pandeng Scholar Program of Shandong Province (tspd20181220), Taishan Young Scholar Program of Shandong Province (tsqn20161065 and tsqn201812129), Youth Top-Talent Project of National Ten Thousand Talents Plan, and the Qilu Young Scholar Program.

## Conflict of Interest

The authors declare that the research was conducted in the absence of any commercial or financial relationships that could be construed as a potential conflict of interest.

## Publisher's Note

All claims expressed in this article are solely those of the authors and do not necessarily represent those of their affiliated organizations, or those of the publisher, the editors and the reviewers. Any product that may be evaluated in this article, or claim that may be made by its manufacturer, is not guaranteed or endorsed by the publisher.

## Supplementary Material

Supplementary Figure 1 Development webserver of predicting nomogram model.

Supplementary Table 1 The baseline characteristics of the survivor cohort and the non-survivor cohort in the training cohort.

Supplementary Table 2 The demographics and clinical characteristics of the validation cohort and the training cohort.

## Abbreviations

AHF: acute heart failure
AHFU: acute Heart Failure Unit
AKI: acute kidney injury
ALT: alanine transaminase
APACHE II: acute Physiology Assessment and Chronic Health Evaluation II
AST: aspartate aminotransferase
BUN: blood urea nitrogen
C-index: the Harrell concordance index
CAD: coronary artery disease
CIs: confidence intervals
CKD: chronic kidney disease
CIC: clinical impact curve
CPR: cardiopulmonary resuscitation
CRRT: continuous renal replacement therapy
DBP: diastolic blood pressure
DCA: decision curve analysis
DM: diabetes mellitus
DN: diabetic nephropathy
ICU: intensive care unit
IMV: invasive mechanical ventilation
IQR: interquartile range
MAP: mean arterial pressure
MEWS: modified early warning score
MIMIC-III: the medical information mart for intensive care III
MV: mechanical ventilation
NEU%: neutrophils ratio
NT-proBNP: N-terminal precursor B-type diuretic peptide
NRI: Net Reclassification Index
ORs: odds ratios
RRT: renal replacement therapy
ROC: receiver-operating characteristic curve
SAPS II: Simplified Acute Physiologic Score II
SBP: systolic blood pressure
SNS: sympathetic nervous system
WBC: white blood cell.
