# Supplementary figures and images for: Development and Validation of a Simple-to-Use Nomogram for Predicting In-Hospital Mortality in Patients With Acute Heart Failure Undergoing Continuous Renal Replacement Therapy
Source: Front Med (Lausanne). 2021 Nov 3;8:678252. doi: 10.3389/fmed.2021.678252 (PMC8595094; doi:10.3389/fmed.2021.678252)

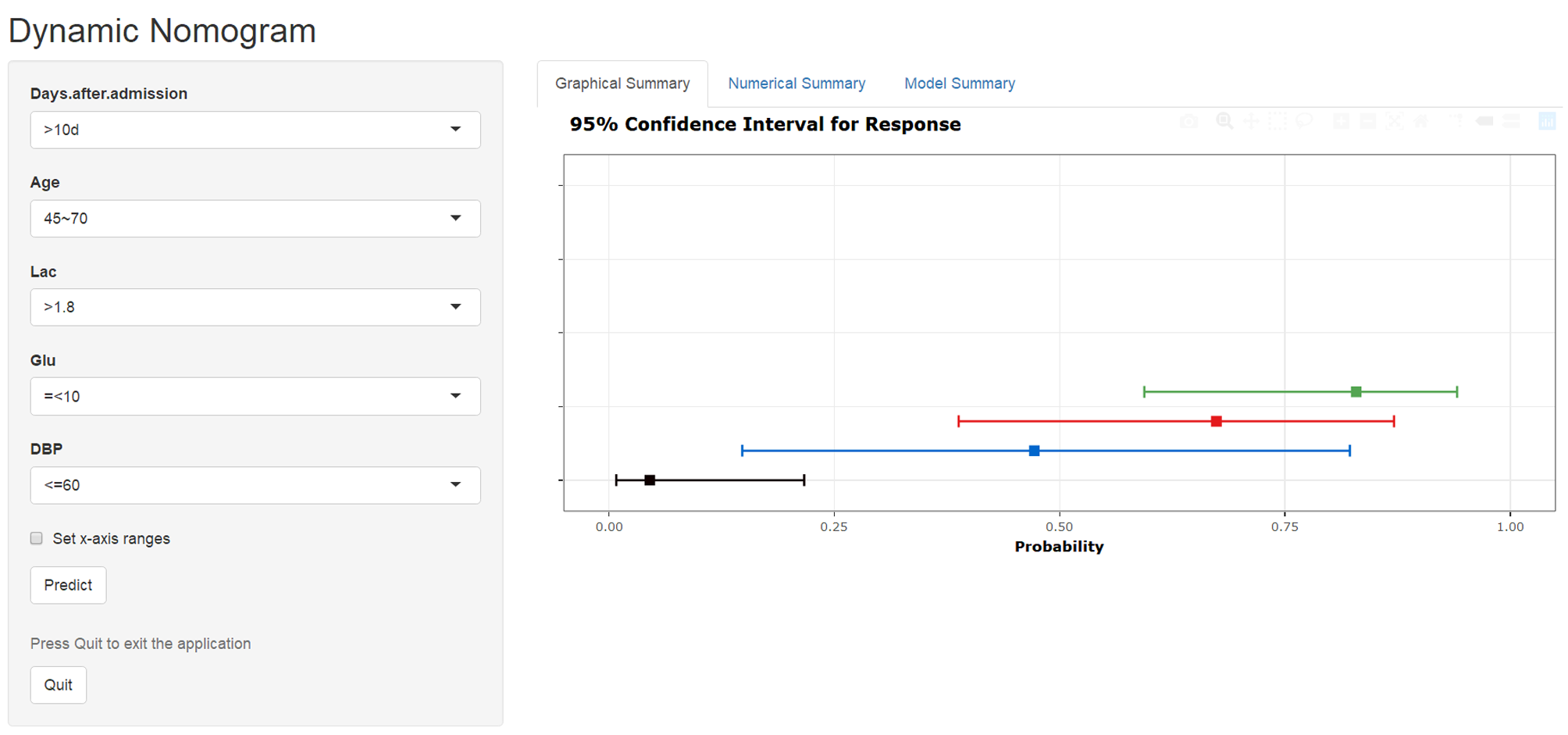

Supplement: Supplementary Figure 1 — Development webserver of predicting nomogram model. [file Image_1.TIF]
