# Supplementary material for: Development and Validation of a Simple-to-Use Nomogram for Predicting In-Hospital Mortality in Patients With Acute Heart Failure Undergoing Continuous Renal Replacement Therapy
Source: Front Med (Lausanne). 2021 Nov 3;8:678252. doi: 10.3389/fmed.2021.678252 (PMC8595094; doi:10.3389/fmed.2021.678252)
Supplement: Supplementary Table 1 — The baseline characteristics of the survivor cohort and the non-survivor cohort in the training cohort. [file Table_1.DOCX]

**Table S1 The baseline characteristics of the Survivor cohort and the Non survivor cohort** **in the training cohort**

|  | Overall | Survivor | Non survivor | *P* value |
| --- | --- | --- | --- | --- |
| n | 159 | 92 | 67 |  |
| sex = Female (%) | 70 (44.0) | 41 (44.6) | 29 (43.3) | 1 |
| age(%) |  |  |  | <0.001 |
| <45 | 19 (11.9) | 16 (17.4) | 3 (4.5) |  |
| 45~70 | 76 (47.8) | 52 (56.5) | 24 (35.8) |  |
| >70 | 64 (40.3) | 24 (26.1) | 40 (59.7) |  |
| DM (%) | 78 (49.1) | 41 (44.6) | 37 (55.2) | 0.243 |
| Hypertension (%) | 70 (44.0) | 39 (42.4) | 31 (46.3) | 0.746 |
| CAD (%) | 84 (52.8) | 44 (47.8) | 40 (59.7) | 0.187 |
| CKD (%) | 94 (59.1) | 57 (62.0) | 37 (55.2) | 0.491 |
| DN (%) | 50 (31.4) | 28 (30.4) | 22 (32.8) | 0.882 |
| CPR (%) | 13 (8.2) | 4 (4.3) | 9 (13.4) | 0.076 |
| MV (%) |  |  |  | 0.262 |
| without MV | 73 (45.9) | 47 (51.1) | 26 (38.8) |  |
| non IMV | 32 (20.1) | 18 (19.6) | 14 (20.9) |  |
| IMV | 54 (34.0) | 27 (29.3) | 27 (40.3) |  |
| T ≤35℃ or >38.5℃ (%) | 12 (7.5) | 9 (9.8) | 3 (4.5) | 0.242 |
| Heart Rate(%) |  |  |  | 0.238 |
| <90 beats/min | 85 (53.5) | 47 (51.1) | 38 (56.7) |  |
| 90-140 beats/min | 70 (44.0) | 41 (44.6) | 29 (43.3) |  |
| >140 beats/min | 4 (2.5) | 4 (4.3) | 0 (0.0) |  |
| Respiration(%) |  |  |  | 1 |
| <20 breaths/min | 75 (47.2) | 43 (46.7) | 32 (47.8) |  |
| 20-30 breaths/min | 75 (47.2) | 44 (47.8) | 31 (46.3) |  |
| ≥30 breaths/min | 9 (5.7) | 5 (5.4) | 4 (6.0) |  |
| SBP ≤120 mmHg (%) | 97 (61.0) | 48 (52.2) | 49 (73.1) | 0.012 |
| DBP ≤60mmHg (%) | 82 (51.6) | 37 (40.2) | 45 (67.2) | 0.001 |
| MAP ≤70mmHg (%) | 51 (32.1) | 24 (26.1) | 27 (40.3) | 0.085 |
| SpO2 (%) |  |  |  | 0.047 |
| ≥99% | 88 (55.3) | 53 (57.6) | 35 (52.2) |  |
| 95-98% | 48 (30.2) | 31 (33.7) | 17 (25.4) |  |
| ≤94% | 23 (14.5) | 8 (8.7) | 15 (22.4) |  |
| Urine volume/h(%) |  |  |  | 0.082 |
| >50 ml/h | 35 (22.0) | 26 (28.3) | 9 (13.4) |  |
| 30~50 ml/h | 31 (19.5) | 17 (18.5) | 14 (20.9) |  |
| <30 ml/h | 93 (58.5) | 49 (53.3) | 44 (65.7) |  |
| WBC > 10*10^9/L (%) | 83 (52.2) | 44 (47.8) | 39 (58.2) | 0.257 |
| NEU% >75% (%) | 98 (61.6) | 55 (59.8) | 43 (64.2) | 0.691 |
| Hemoglobin >90 g/L (%) | 77 (48.4) | 49 (53.3) | 28 (41.8) | 0.205 |
| Platelet ≤130*10^9/L (%) | 54 (34.0) | 27 (29.3) | 27 (40.3) | 0.204 |
| Potassium <3.5 or >5.5 mmol/L(%) | 33 (20.8) | 15 (16.3) | 18 (26.9) | 0.155 |
| Sodium <137 or >147 mmol/L(%) | 93 (58.5) | 51 (55.4) | 42 (62.7) | 0.451 |
| Calcium <2.0 or >2.6mmol/L (%) | 71 (44.7) | 40 (43.5) | 31 (46.3) | 0.851 |
| ALT >40U/L (%) | 60 (37.7) | 32 (34.8) | 28 (41.8) | 0.463 |
| AST >60U/L (%) | 68 (42.8) | 32 (34.8) | 36 (53.7) | 0.026 |
| Creatinine >430umol/L (%) | 58 (36.5) | 43 (46.7) | 15 (22.4) | 0.003 |
| BUN >20mmol/L(%) | 95 (59.7) | 56 (60.9) | 39 (58.2) | 0.862 |
| Blood glucose >10mmol/L (%) | 45 (28.3) | 18 (19.6) | 27 (40.3) | 0.007 |
| Lactic acid >1.8mmol/L (%) | 66 (41.5) | 29 (31.5) | 37 (55.2) | 0.005 |
| Days after admission (%) |  |  |  | <0.001 |
| ≤3d | 76 (47.8) | 56 (60.9) | 20 (29.9) |  |
| 4~10d | 52 (32.7) | 27 (29.3) | 25 (37.3) |  |
| >10d | 31 (19.5) | 9 (9.8) | 22 (32.8) |  |
| MEWS (mean±SD) | 3.03±2.04 | 2.85±2.12 | 3.28±1.91 | 0.184 |
| SUPER.Score (mean±SD) | 3.31±1.55 | 3.11±1.61 | 3.60±1.41 | 0.049 |
