# Supplementary material for: Development and Validation of a Simple-to-Use Nomogram for Predicting In-Hospital Mortality in Patients With Acute Heart Failure Undergoing Continuous Renal Replacement Therapy
Source: Front Med (Lausanne). 2021 Nov 3;8:678252. doi: 10.3389/fmed.2021.678252 (PMC8595094; doi:10.3389/fmed.2021.678252)
Supplement: Supplementary Table 2 — The demographics and clinical characteristics of the validation cohort and the training cohort. [file Table_2.DOCX]

**Table S2 The demographics and clinical characteristics of the Validation cohort and the Training cohort**

|  | Overall | Validation cohort | Training Cohort | *P* test |
| --- | --- | --- | --- | --- |
| n | 226 | 67 | 159 |  |
| Sex = Male (%) | 123 (54.4) | 34 (50.7) | 89 (56.0) | 0.566 |
| group = survivor (%) | 128 (56.6) | 36 (53.7) | 92 (57.9) | 0.671 |
| age(%) |  |  |  | 0.234 |
| <45 | 23 (10.2) | 4 (6.0) | 19 (11.9) |  |
| >70 | 98 (43.4) | 34 (50.7) | 64 (40.3) |  |
| 45~70 | 105 (46.5) | 29 (43.3) | 76 (47.8) |  |
| Non-DM (%) | 113 (50.0) | 32 (47.8) | 81 (50.9) | 0.771 |
| Non-Hypertension (%) | 123 (54.4) | 34 (50.7) | 89 (56.0) | 0.566 |
| Non-CAD (%) | 105 (46.5) | 30 (44.8) | 75 (47.2) | 0.854 |
| Non-CKD (%) | 94 (41.6) | 29 (43.3) | 65 (40.9) | 0.852 |
| Non-DN (%) | 158 (69.9) | 49 (73.1) | 109 (68.6) | 0.598 |
| Non-CPR (%) | 205 (90.7) | 59 (88.1) | 146 (91.8) | 0.523 |
| MV (%) |  |  |  | 0.411 |
| without MV | 99 (43.8) | 26 (38.8) | 73 (45.9) |  |
| IMV | 83 (36.7) | 29 (43.3) | 54 (34.0) |  |
| non IMV | 44 (19.5) | 12 (17.9) | 32 (20.1) |  |
| Temperature=35~38.5℃ (%) | 203 (89.8) | 56 (83.6) | 147 (92.5) | 0.076 |
| Heart Rate(%) |  |  |  | 0.633 |
| <90 beats/min | 122 (54.0) | 37 (55.2) | 85 (53.5) |  |
| >140 beats/min | 7 (3.1) | 3 (4.5) | 4 (2.5) |  |
| 90-140 beats/min | 97 (42.9) | 27 (40.3) | 70 (44.0) |  |
| Respiration(%) |  |  |  | 0.573 |
| <20 breaths/min | 103 (45.6) | 28 (41.8) | 75 (47.2) |  |
| ≥30 breaths/min | 15 (6.6) | 6 (9.0) | 9 (5.7) |  |
| 20-30 breaths/min | 108 (47.8) | 33 (49.3) | 75 (47.2) |  |
| SBP >120mmHg (%) | 81 (35.8) | 19 (28.4) | 62 (39.0) | 0.17 |
| DBP >60mmHg (%) | 107 (47.3) | 30 (44.8) | 77 (48.4) | 0.722 |
| MAP >70mmHg (%) | 149 (65.9) | 41 (61.2) | 108 (67.9) | 0.411 |
| SpO2 (%) |  |  |  | 0.083 |
| <94% | 39 (17.3) | 16 (23.9) | 23 (14.5) |  |
| ≥99% | 115 (50.9) | 27 (40.3) | 88 (55.3) |  |
| 94-98% | 72 (31.9) | 24 (35.8) | 48 (30.2) |  |
| Urine volume/h(%) |  |  |  | 0.724 |
| <30ml/h | 136 (60.2) | 43 (64.2) | 93 (58.5) |  |
| >50ml/h | 48 (21.2) | 13 (19.4) | 35 (22.0) |  |
| 30~50ml/h | 42 (18.6) | 11 (16.4) | 31 (19.5) |  |
| WBC > 10*10^9/L (%) | 117 (51.8) | 34 (50.7) | 83 (52.2) | 0.957 |
| NEU% >75% (%) | 141 (62.4) | 43 (64.2) | 98 (61.6) | 0.834 |
| Hemoglobin >90 g/L (%) | 121 (53.5) | 39 (58.2) | 82 (51.6) | 0.443 |
| Platelet >130*10^9/L (%) | 151 (66.8) | 46 (68.7) | 105 (66.0) | 0.82 |
| Potassium 3.5~5.5 mmol/L(%) | 169 (74.8) | 43 (64.2) | 126 (79.2) | 0.027 |
| Sodium = 137~147 mmol/L (%) | 93 (41.2) | 27 (40.3) | 66 (41.5) | 0.983 |
| Calcium = 2.0~2.6 mmol/L (%) | 130 (57.5) | 42 (62.7) | 88 (55.3) | 0.383 |
| ALT >40 U/L (%) | 87 (38.5) | 27 (40.3) | 60 (37.7) | 0.832 |
| AST >60 U/L (%) | 101 (44.7) | 33 (49.3) | 68 (42.8) | 0.454 |
| Creatinine>430umol/L (%) | 85 (37.6) | 27 (40.3) | 58 (36.5) | 0.696 |
| BUN >20mmol/L(%) | 138 (61.1) | 43 (64.2) | 95 (59.7) | 0.635 |
| Blood glucose >10mmol/L (%) | 68 (30.1) | 23 (34.3) | 45 (28.3) | 0.457 |
| Lactic acid>1.8mmol/L (%) | 92 (40.7) | 26 (38.8) | 66 (41.5) | 0.818 |
| Days after admission before CRRT (%) |  |  |  | 0.97 |
| ≤3d | 107 (47.3) | 31 (46.3) | 76 (47.8) |  |
| >10d | 44 (19.5) | 13 (19.4) | 31 (19.5) |  |
| 4~10d | 75 (33.2) | 23 (34.3) | 52 (32.7) |  |
| MEWS (mean±SD) | 3.16±2.02 | 3.48±1.96 | 3.03 (2.04) | 0.13 |
| SUPER.Score (mean±SD) | 3.45±1.59 | 3.78 (1.66) | 3.31 (1.55) | 0.046 |
